# The effectiveness of telehealth gait retraining in addition to standard physical therapy treatment for overuse knee injuries in soldiers: a protocol for a randomized clinical trial

**DOI:** 10.1186/s13063-023-07502-x

**Published:** 2023-10-16

**Authors:** Michael S. Crowell, Richard A. Brindle, Erin M. Miller, Nicholas Reilly, Kevin R. Ford, Donald L. Goss

**Affiliations:** 1 Baylor University – Keller Army Community Hospital Division 1 Sports Physical Therapy Fellowship, 900 Washington Road, West Point, NY 10966 USA; 2 Shaw Sports Turf, Shaw Industries Group, Inc., Dalton, GA USA; 3 Department of Physical Therapy, High Point University, High Point, NC USA

**Keywords:** Running injury, Running biomechanics, Rehabilitation

## Abstract

**Introduction:**

Running is the most common cardiovascular exercise in the military. However, there is a high incidence of running-related overuse injuries that reduces military readiness. Gait retraining is a common intervention to treat running-related injuries, but the high cost of equipment and lack of clinician expertise and availability reduces utilization. Gait retraining intervention in a telehealth format might improve feasibility. The purpose of this randomized clinical trial is to determine the effectiveness of a telehealth gait retraining intervention on pain, self-reported function, and biomechanical risk factors for injury in service members who present to a Military Health System physical therapy clinic with an overuse knee injury.

**Methods:**

This is a parallel, two-arm, single-blind randomized clinical trial. The two independent variables are intervention (2 levels: telehealth gait retraining intervention with standard of care or only standard of care) and time (3 levels: baseline, 10 weeks or post-intervention, 14 weeks). Participants between the ages of 18 to 60 years will be included if they report knee pain during and/or after running to be anywhere from a 3 to a 7 on the numerical pain rating scale and demonstrate a rearfoot strike pattern. The primary dependent variables are as follows: (1) pain (worst pain during and/or after running) and (2) foot strike pattern (conversion rate from rearfoot to non-rearfoot foot strike pattern during running). Secondary outcomes include patient self-reported function and running biomechanics.

**Discussion:**

The effectiveness of a telehealth gait retraining intervention to reduce pain and modify foot strike pattern is not known. The results of this study may help determine the effectiveness and feasibility of a telehealth gait retraining intervention to reduce pain, change foot strike, improve function, and improve running gait biomechanics.

**Trial registration:**

ClinicalTrials.gov, NCT04269473. Registered 05 February 2020.

**Supplementary Information:**

The online version contains supplementary material available at 10.1186/s13063-023-07502-x.

## Background

Soldiers need to possess excellent muscular strength and cardiovascular endurance to perform military duties well. Running is the most common mode of exercise for soldiers to maintain cardiovascular fitness. However, running-related overuse injuries reduce military readiness, result in lost duty time, and cost money. Running was attributed to approximately 30% of all injuries sustained by soldiers [1, 2] and approximately 50% of all sports, exercise, or recreational activity-related injuries sustained by soldiers [3]. The rate of overuse injuries associated with running in soldiers is similar to that of civilian populations, where 19% to 79% of recreational runners incur an overuse injury each year [4].

In both military and civilian runners, the knee is the site of most running injuries [3–6]. The presence of knee pain reduces military readiness as soldiers are either unable to complete or have limited capacity to complete basic military tasks that load the knee [7, 8]. Unfortunately, chronic symptoms are common after running injuries. At long-term follow-up, approximately half of atraumatic knee pain patients report persistent pain and reduced function [9–11]. Recurrence is also common after running injuries affecting the knee [12] and a prior history of injury is a recognized risk factor for running injuries in both civilian and military populations [13–15]. Optimal treatment strategies for overuse knee injuries in are needed to avoid subsequent injury, restore running ability, and return to duty as quickly as possible.

Optimal treatment of musculoskeletal injuries due to running may reduce the risk of recurrence and improve return to duty rates. Treatment for knee pain during running has typically involved strengthening and stretching, but these interventions do not adequately address running biomechanics [16], a common underlying cause of running-related knee injuries [17]. Gait retraining interventions have been effective at inducing specific changes to running biomechanics with long-term effects [18, 19]. A comprehensive approach to running-related injury treatment combining a gait retraining intervention with a standard of care strengthening and stretching intervention may improve injury recurrence and return to duty rates in soldiers [20].

Gait retraining interventions are complex treatments based on motor control principles to alter an individual’s running biomechanics. Substantial enhancements to injury prevention and rehabilitation outcomes in athletes were reported by multiple research studies using gait retraining [18, 21, 22]. In military populations, improvements in pain, function, and occupational readiness as well as reductions in healthcare cost and injury rates following gait retraining were reported [20, 23, 24]. Large group running gait retraining during basic military training resulted in significantly fewer trainees both removed from training and separated from service because of injury [20]. Specifically, gait retraining to change foot strike pattern from a rearfoot strike (RFS) to a non-rearfoot strike (NRFS) has been shown to improve function, pain, and known biomechanical factors related to running knee injury to include patellofemoral joint stress and components of the vertical ground reaction force [25–27]. However, most gait retraining interventions require multiple clinic visits [17] which limits feasibility in physical therapy clinics in the Military Health System.

With increasing technological capabilities, clinicians and researchers have sought new ways to leverage consumer technology for telehealth interventions. Administering a gait retraining intervention in a telehealth format might improve feasibility through decreased cost and time demands of treatment compared to the traditional in-clinic format. Recently, a biomechanical risk factor for overuse injury, high instantaneous vertical loading rates, was successfully decreased in healthy runners using a telehealth gait retraining intervention to increase step rate [28]. However, increasing habitual running step rate may only be suitable for runners with greater loading rates, as no change in loading rates was noted with increasing step rate in runners with increasing step rate in runners with low loading rates [29].

Other than step rate, adaptation of a non-rearfoot strike pattern in runners with a rearfoot strike pattern may be an effective and feasible treatment for overuse knee injuries. Gait retraining interventions focused on foot strike pattern may be more suitable for patients with an overuse knee injury than those focused on step rate. Although there are greater decreases in loading rates in healthy runners who convert from a habitual rearfoot strike pattern to a non-rearfoot strike pattern compared to increasing step rate [29, 30], pain is often the greatest concern in patients with overuse knee injuries. While an in-clinic gait retraining program that transitions runners to a non-rearfoot strike pattern is effective in reducing pain during and/or after running, this intervention has not been studied in a telehealth format. Therefore, the effectiveness of a telehealth gait retraining intervention to reduce pain by adopting a non-rearfoot strike pattern during running on biomechanical and clinical outcomes in patients with overuse knee injuries is unknown.

The purpose of this randomized clinical trial is to determine the effectiveness of a telehealth gait retraining intervention on pain during/after running, foot-strike, self-reported function, and biomechanical risk factors for injury in soldiers who present to a Military Health System physical therapy clinic with an overuse knee injury. The primary objectives are to assess the effect of telehealth gait retraining on pain during and/or after running and observed foot strike pattern during running. We hypothesize that participants who receive the telehealth gait retraining intervention in addition to standard of care will report greater improvements in self-reported pain intensity and will demonstrate a greater proportion of participants who display an NRFS running gait pattern than those who receive standard of care alone. Additional objectives are to assess the effect of telehealth gait retraining on self-reported function and gait biomechanics. We hypothesize that participants who receive the telehealth gait retraining intervention will report reduced pain during/after running, transition from a rearfoot to a non-rearfoot strike pattern, will report greater improvements in function, and will demonstrate improved gait biomechanics (reduced foot strike index, foot angle, step rate, stride length, ground contact time, peak hip adduction angle, peak knee adduction angle, knee stiffness, peak rearfoot inversion moment, and vertical loading rates) during running.

## Methods

### Trial design

**Fig. 1:**
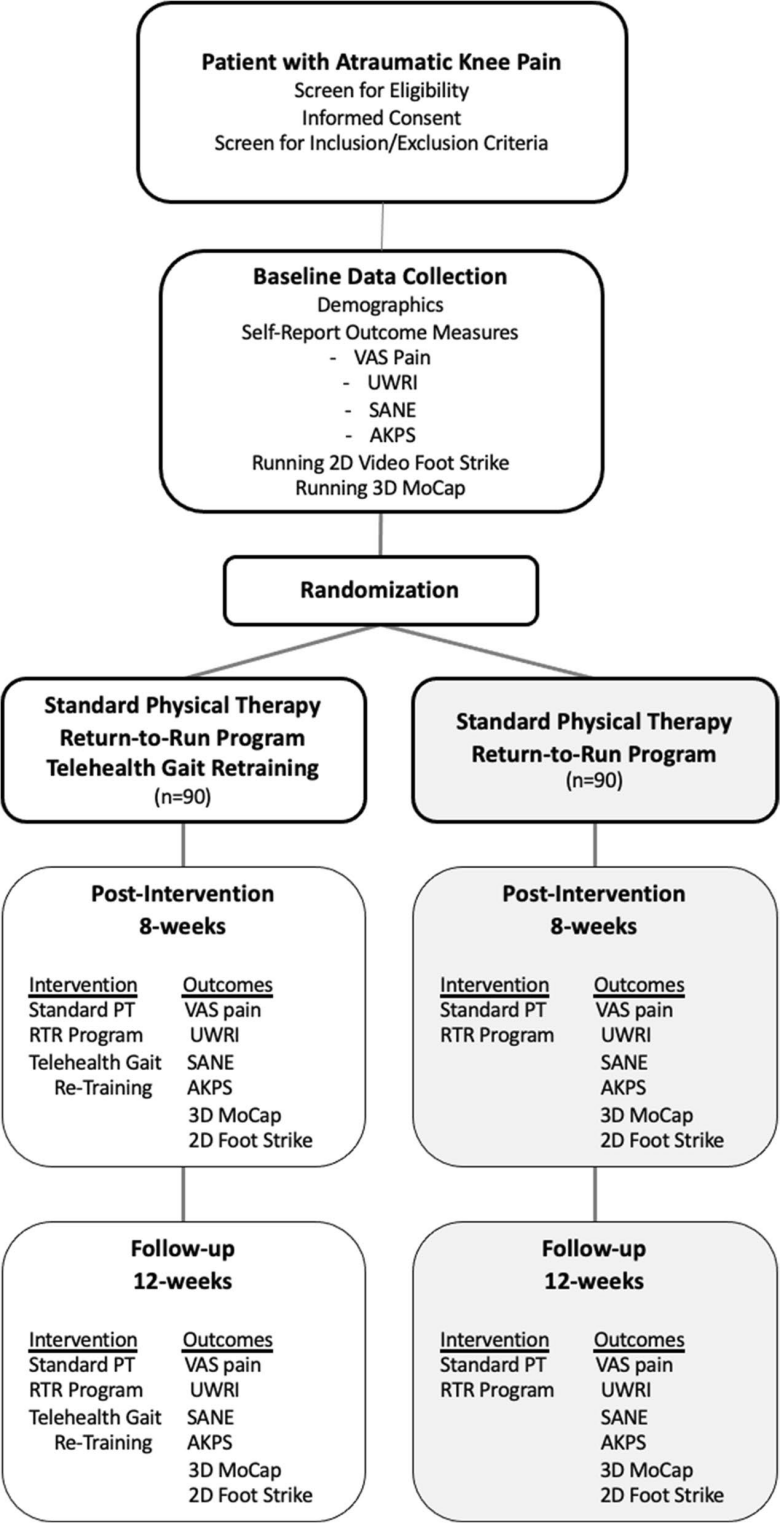
Proposed recruitment flow for the study along with interventions performed and outcomes collected at each timepoint. PT, physical therapy; RTR, return-to-run; VAS, visual analog scale; UWRI, University of Wisconsin Running Injury and Recovery Index; SANE, Single Assessment Numerical Evaluation; AKPS, Anterior Knee Pain Scale; 3D, three-dimensional; 2D, two-dimensional; Mocap, motion capture

This study will be a parallel, two-arm, single-blind randomized clinical superiority trial with 1:1 allocation ratio. Participants will complete three data collection sessions before, immediately after, and 1 month after the intervention. Participants will receive either the telehealth gait retraining intervention with standard of care or only standard of care over 8 weeks. During the 8-week intervention, all participants will follow-up at the discretion of their physical therapist (typically every 2 weeks), perform a rehabilitation program that includes a home exercise program with or without supervised clinic sessions, and complete a standard return-to-run program that systematically progresses the duration, frequency, and intensity of running. Participants receiving the telehealth gait retraining intervention will also receive feedback on their running form via a smart device application. The flow of participants throughout this study is shown in Fig. 1.

The 2013 Standard Protocol Items: Recommendations for Interventional Trials (SPIRIT) was followed in designing this clinical trial (Supplemental Materials) [31]. The Consolidated Standards of Reporting Trials (CONSORT) statement and Template for Intervention Description and Replication (TIDieR) checklist will be followed in reporting the results of this clinical trial [32, 33].

### Participants and study setting

This randomized clinical trial will be a dual-site study. A total of 180 participants will be recruited for this study. Participants who seek treatment for atraumatic knee pain will be recruited from the cadet and active-duty soldier population at Keller Army Community Hospital, United States Military Academy, West Point, NY, or Womack Army Medical Center, Fort Liberty, NC.

To participate in this study, service members must be Department of Defense beneficiaries between the ages of 18 to 60 years, report knee pain during and/or after running to be anywhere from a 3 to a 7 on the numerical pain rating scale (NPRS), and demonstrate a rearfoot strike pattern. Potential participants who demonstrate a non-rearfoot strike pattern, have a concurrent lower extremity injury or evidence of knee internal derangement (i.e., ligamentous instability or meniscal pathology), lack adequate lower extremity strength, have a running-limiting profile for something other than knee pain, have a diagnosed rheumatoid or neurological disease, are pregnant, or are deploying or moving within four months of consent will be excluded from the study. Lower extremity strength will be assessed by the participant’s ability to perform at least 10 consecutive single leg squats, bilaterally and at least 20 consecutive single-leg heel raises, bilaterally. Foot strike pattern will be assessed during a treadmill run at a self-selected speed that the participant would run at for an easy 20-min run [34]. After participants run for 3 min, a 10-s video of their feet in the sagittal plane will be recorded at 240 Hz on mobile device (Apple Inc., Cupertino, CA, USA) inside the OnForm: Video Analysis application (OnForm, Inc., Fort Collins, CO, USA). An investigator blinded to the treatment group will determine foot strike pattern by reviewing recorded footage of 10 s of consecutive steps of the left and right feet [35]. A rearfoot strike pattern will be defined contacting the ground with the posterior third of the shoe during the majority of steps over 10 s [35].

Only participants who meet the screening criteria will be enrolled in the study. The baseline data collection will include a running-related injury history questionnaire and report their worst pain experienced during and/or after running in the past 7 days on visual analog scales (VAS) [36, 37]. Additionally, participants will report their perceived knee function, condition, and running ability by completing the Anterior Knee Pain Scale (AKPS) [38], Single Assessment Numerical Evaluation (SANE) [39, 40], and the University of Wisconsin Running Injury and Recovery Index (UWRI) [41, 42], respectively. After completing all surveys, participants’ three-dimensional running biomechanics will be measured using a force plate instrumented treadmill and motion capture system.

### Randomization and blinding

Following the baseline data collection, participants will be randomly assigned by a study investigator to either the control group or the experimental group using a concealed allocation process. An investigator will not reveal group allocation until all baseline measurements have been completed. The participant’s group assignment will be recorded with a unique participant identifier until completion of all data collection or through the final follow-up. Creation of the randomization sequence and concealment of participant group assignments will be completed by an individual not involved with the current study. The randomization sequence will utilize a random permuted block approach with a 1:1 allocation ratio to keep control and experimental group size similar throughout the data collection process [43]. The investigator determining foot strike pattern and the physical therapist providing standard physical therapy will be blinded to group allocation. The participant, physical therapist performing the telehealth gait retraining intervention, and investigators administering self-report surveys and collecting biomechanics data will be aware of the participant’s treatment group. Unblinding will only be permissible in cases where the participant must be disenrolled from the study due to adverse effects.

## Interventions

### Standard physical therapy (active control group and study intervention group)

**Fig. 2:**
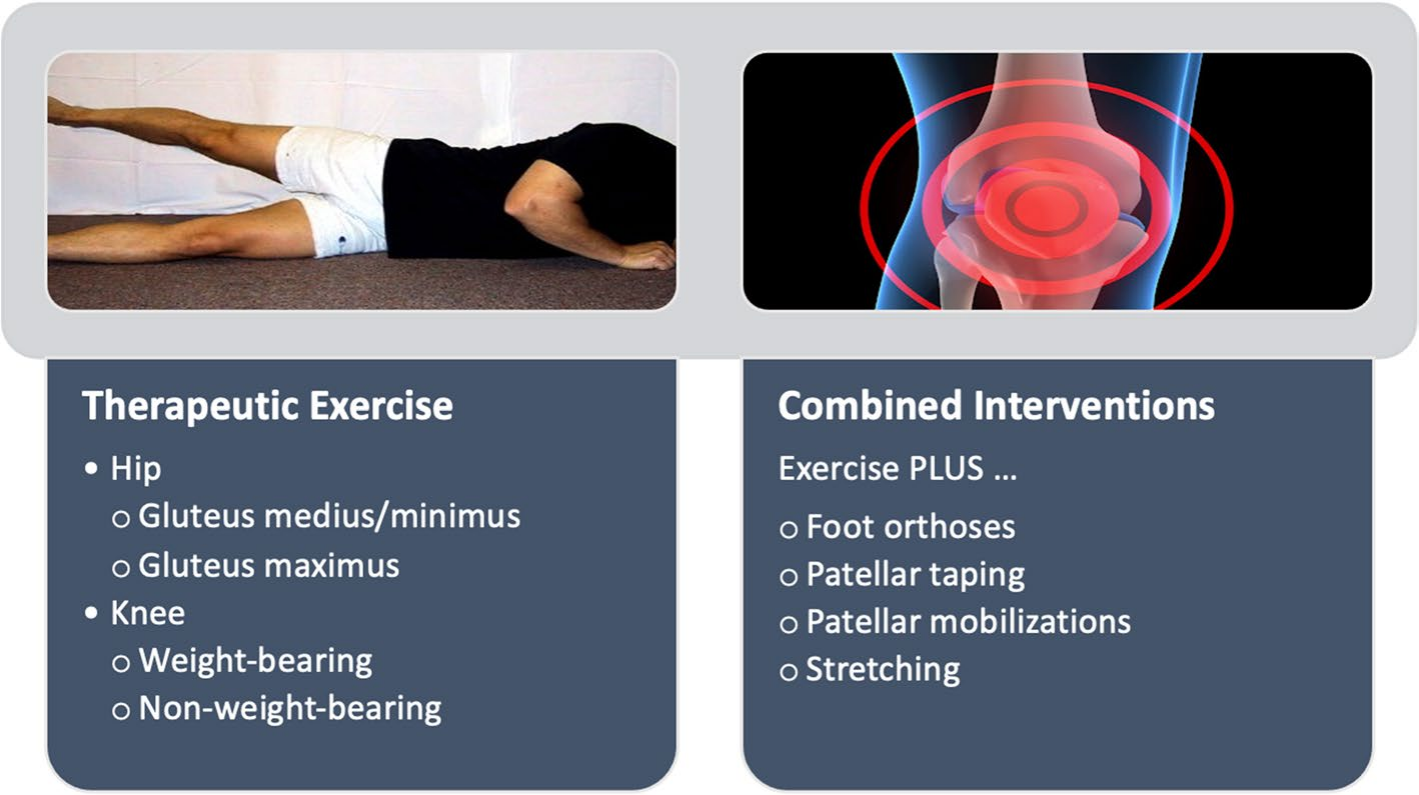
Components of a standard physical therapy treatment for atraumatic knee pain

During treatment, participants in both the control and experimental groups will receive standard of care physical therapy treatment that consists of a home exercise program with or without additional supervised clinic visits [44]. Participants will follow-up at the discretion of their referring physical therapist, or approximately every 2 weeks. Potential elements of the standard physical therapy treatment for overuse knee injuries are shown in Fig. 2 [44].

A supplemental exercise program for both the control group and intervention group will also be employed to target muscles that are utilized more extensively when running with a non-rearfoot strike. The exercises consist of lower extremity strength and flexibility exercises for the knee flexors, ankle plantar flexors, and plantar intrinsic foot musculature (Additional file 1).

### Standard return to run program (active control group)

The return-to-run program is a standard prescription of running volume progression (Table 1) designed to allow the participant to adapt to gradually increasing load. Participants in the control group will not receive any gait retraining in the form of verbal or tactile cues, coaching, or instruction to change their running form.

**Table 1:** Standard return-to-run program

Phase	Rate of perceived exertion	Walk interval	Run interval	Total distance
1	3/10 (“easy”)	2 min	3 min	1.0 mile
2	3/10 (“easy”)	2 min	3 min	1.5 miles
3	4/10 (“somewhat easy”)	2 min	4 min	1.5 miles
4	4/10 (“somewhat easy”)	2 min	4 min	2.0 miles
5	5/10 (“somewhat hard”)	1 min	4 min	2.0 miles
6	5/10 (“somewhat hard”)	0 min	All minutes	2.0 miles
1. **Stretch and warm-up** for 10 to 15 min before exercise 2. Have at least ***one day of rest*** in between each run 3. Try each phase **at least TWICE** before progressing to the next phase. **IF** you experience *swelling, stiffness, or an increase in pain* during and/or after running, **DO NOT** progress to the next phase. Stay in the phase **until** you can complete it **without** swelling, stiffness, or an increase in pain 4. Perform at the defined rate of perceived exertion **AND** on **level surfaces – NO HILLS** 5. Use good jogging shoes that are **no more than 6 months old**				

### Return to run program with telehealth gait retraining (study intervention group)

Participants in the intervention group will receive telehealth gait retraining instruction on how to transition from a rearfoot strike pattern to a non-rearfoot strike pattern during their progression through the standard return-to-run program. Feedback will be facilitated in OnForm software application accessible via the participants’ personal mobile device. Participants in the intervention group will be asked to record a 10-s video of themselves running on a treadmill from the sagittal plane view. To standardize the view of each video, the participants will be instructed to ensure that the video: aligns the bottom of the screen with the treadmill base and holds the mobile device as straight up and down as possible, captures the feet hitting the treadmill belt, and only records video from the waist down. The video will be shared to a therapist who then provides auditory and visual feedback to the video before sending back to the participant.

Therapists will use standardized visual and verbal cues reported by previous interventions which were successful in changing foot strike pattern and increasing step rate in previous studies for their feedback (Additional file 2) [25, 45–47]. While the cues used to change running will be administered at the discretion of the physical therapist, each therapist providing feedback to participants will undergo training to ensure uniformity in use of auditory and visual feedback to decrease the potential effect of therapist on intervention outcome. Participants view the provided feedback in their OnForm account at least twice and once before their next run. Running videos will be recorded and therapist directed feedback will be shared once a week during the first 4 weeks, then again at weeks 6 and 8 of the 8-week intervention. The faded feedback schedule aligns with motor learning theory for new motor skill acquisition and retention [48] and previous gait retraining interventions reported long-term retention with similar faded feedback schedules [19, 23].

There is a minimal risk of bone stress injury of the metatarsals or pain in a region other than the knee with a transition to a non-rearfoot strike pattern [49–51]. To minimize this risk, the participant’s response to running will be assessed weekly for the first 4 weeks and bi-weekly thereafter until study completion. Investigators will record reports of either increases pain in the affected knee joint and/or pain in any other body region. Participants reporting metatarsal pain with running will be evaluated in-person by their primary physical therapist and will be disenrolled from the study if presenting with signs/symptoms of bone stress injury.

## Data collection

At the baseline data collection, participants will change into athletic shorts, short-sleeve shirt, and their normal running shoes. Their height will be measured before preparing for three-dimensional gait analysis. Three-dimensional positions of retroreflective markers will be recorded using a nine-camera motion capture system (Vicon, Oxford, UK) at 120 Hz. Ground reaction forces will be recorded simultaneously and in synchronization with the motion capture system at 1200 Hz via force plates arranged in-series on an instrumented treadmill (AMTI, Watertown, MA, USA).

**Fig. 3:**
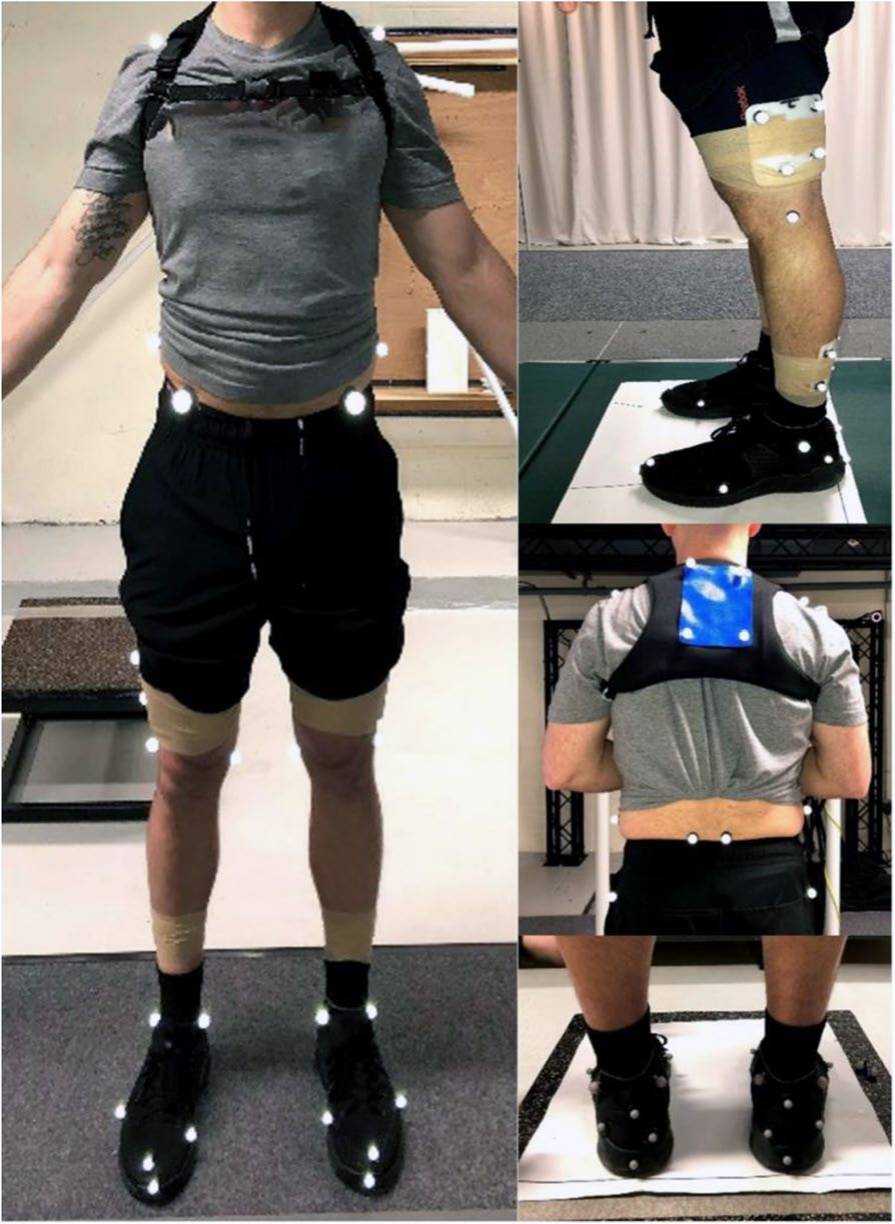
Components of a standard physical therapy treatment for atraumatic knee pain

Retroreflective markers will be positioned on participants’ trunk, pelvis, and lower extremity (Fig. 3). Specifically, markers will be positioned on participants bilateral acromion processes, iliac crests, greater trochanters, medial and lateral femoral epicondyles and malleoli, and first and fifth metatarsal heads to define segmental coordinate systems.

Additionally, local coordinate systems for tracking segments will be defined by markers positioned on bilateral anterior and posterior superior iliac spines to track the pelvis segment and bilateral superior, lateral, and medial shoe heels to track the foot. The trunk’s local coordinate system will be defined by four noncolinear markers on a thermoplastic shell positioned over the thoracic spine via a neoprene vest. Local coordinate systems for the thigh and shank will be defined by four noncolinear markers on thermoplastic shells positioned directly over the distal thighs and proximal shanks and secured using self-adherent wraps. A standing calibration trial will be recorded, after which all markers defining segmental coordinate systems will be removed.

Treadmill speed will be set at a self-selected speed that the participant would run at for an easy 20-min run [52]. After participants run for 6 min, a 20-s motion capture trial will be recorded. Treadmill speed will then be set to a standard speed of 2.68 m/s, and another 20-s motion capture trial will be recorded. After the last week of the intervention period (week 8), all participants will be asked to return to the clinic for the post-intervention data collection. At the post-intervention and follow-up visits, self-reported outcomes will be completed and running biomechanical analysis will be collected from all participants as previously. An additional treadmill speed condition, the speed that was self-selected during the pre-intervention visit, will be collected during the post-intervention and final follow-up data collection visits.

A visual Stroop task will be applied during the follow-up visit to assess retention of the newly learned running gait. The application of the dual-task paradigm requires participants to devote attentional resources to the cognitive task, which limits their ability to actively control their running gait, revealing the degree of motor learning. The visual Stroop task will be projected on a monitor positioned at eye level directly in front of the participant. The words red, blue, green, brown, and purple will be displayed in either with the font color matching the meaning of the word (congruent) or not matching (incongruent) [53]. Color-word stimuli will be presented every 3 s during the recorded running trial, and participants will be instructed to report the font color and not the word meaning. Response time will be automatically recorded via Bluetooth ear pods using SuperLab 6 stimulus presentation software (Cedrus, San Pedro, CA). Running biomechanics will be concurrently recorded while the participant runs at their preferred pace on the treadmill. After the follow-up data collection, participants will be considered complete with the study.

To promote adherence to the treatment protocol, an investigator will email each participant on Monday and Wednesday of each week to remind them to submit their running video. If a participant fails to submit a video for 2 consecutive weeks, they will be considered non-adherent to the treatment and will be withdrawn from the study. In the same bi-weekly email, participants will be reminded to complete their home exercises, continue the return-to-run program, and follow-up with their primary physical therapist every 2 weeks. Participants will also be asked if they have any questions.

To promote retention, an investigator will send an email to each participant 1 week and 48 h prior to the post-intervention data collection point to schedule their follow-up visit and to complete the on-line patient self-report surveys. If the participant does not respond to the initial emails, the investigator will follow-up with a phone call to the participant. The participant will be disenrolled from the study if they do not respond within 1-week past the post-intervention data collection point date.

## Outcome measures

### Primary outcomes

#### Pain

Worst pain during and/or after running will be assessed by a visual analog scale (VAS). The VAS assesses the perception of pain intensity by asking the participant to mark their level of pain along a 100-mm line, where the left limit indicates no pain and the right limit indicates the worst pain imaginable [36, 37]. The VAS is a valid and reliable measure of pain intensity [36, 37, 54, 55].

#### Foot strike pattern

Two-dimensional (2D) sagittal plane running video will be collected while the participant runs on an instrumented treadmill (AMTI, Watertown, MA, USA) using an Apple iPad Pro (Apple, Cupertino, CA) sampling at 240 Hz (Hz) to assess foot strike pattern (FSP). The FSP utilized for the majority of 10 s of the third running trial will used to categorize participants’ FSPs dichotomously into 1 of 2 groups, rearfoot strike (RFS) or non-rearfoot strike (NRFS). A RFS will be considered initial plantar contact observed in the posterior one third of the foot. A NRFS will be considered initial plantar contact observed in the anterior two thirds of the foot. If both anterior and posterior aspects of the foot make initial contact with the ground simultaneously, the foot strike will be considered an NRFS pattern. This method has previously demonstrated excellent inter- and intra-rater reliability and validity when compared with plantar pressure insoles [35].

### Secondary outcomes

#### Patient self-reported function

Each participant will complete assessments of function with the AKPS, the SANE, and the UWRI. The AKPS consists of 13 questions about a variety of running related tasks and knee function, such as limping, walking, running, jumping, stairs, knee swelling, and pain. Scores range from 0 to 100, with lower scores indicating greater disability. The AKPS is a reliable and responsive measure of function in patients with anterior knee pain, with a minimal detectable change of 14 points [38]. The SANE is a global rating scale that is a valid and responsive tool for measuring knee function. The SANE is scored on a 0–100% scale with 0% equating to unable to function and 100% equaling full function [39, 40, 56]. The MCID of the SANE for lower extremity conditions has been reported as 7% at 6-month follow-up. The UWRI is a running-specific patient reported outcome measure and is scored on a scale from 0 to 36 with 36 equaling full running function and a score of 0 equating to inability to run. The UWRI is reliable, valid, and responsive to change after a running-related injury, with measurement properties superior to other patient reported outcome measures typically used in this population. The MCID for the UWRI is 8 points [41, 42].

#### Running biomechanics

Marker trajectories will be filtered using a low-pass second-order Butterworth filter with a cutoff frequency of 12 Hz. Kinetic and kinematic analyses were performed based on the Plug-In Gait model (Vicon Nexus 1.8.2, Oxford Metrics, UK). Variables of interest were calculated using Visual 3D (C-Motion Bethesda MD) and extracted using custom processing software (Matlab, MathWorks, Natick MA). Temporal spatial variables of interest, kinematic, and kinetic data will be collected during gait using a 16-camera, three-dimensional motion capture system as each participant runs on a force-plate instrumented treadmill, respectively. Variables of interest are step rate and ground contact time and will be calculated from the force plate signal. Foot contact and toe off events will be defined as when the vertical ground reaction force exceeds and falls below a 50-N threshold, respectively. Step rate is the number of foot contacts per minute, contact time is the time between foot contact and toe off events, and step length is the distance between the proximal contralateral and ipsilateral foot segments at their respective foot contact events plus the distance traveled by the treadmill belt. Kinematic variables of interest are peak knee adduction angle and peak hip adduction angle. Hip and knee angles were calculated using rigid body principles and the joint coordinate system with an XYZ order of rotation [57]. Peak hip adduction angle was the maximum frontal plane hip angle during the first 60% of the stance phase. Peak knee adduction angle was the maximum frontal plane knee angle during the first 60% of the stance phase. Kinetic variables of interest include average vertical loading rate, instantaneous vertical loading rate, knee stiffness, and peak rearfoot inversion moment. Average vertical loading rate was defined in two ways: (1) the average slope from 20 to 80% of the vGRF magnitude at the impact peak and (2) the average slope from 3 to 12% of stance time [58]. Data from the entire 20-s trial on the left and the right of the running trial will be analyzed. Three-dimensional motion capture data will be processed with all kinematic variables being extracted using Visual 3D (C-Motion, Germantown, MD, USA) whereas kinetic variables derived from vertical ground-reaction force of interest will be processed and extracted using custom code written in Matlab (MathWorks, Natick, MA, USA).

## Data analysis

An a priori power analysis was based on previously published data by Roper et al. [25] for the between group difference in pain during/after running, which reported a large effect size. We conservatively adjusted to a moderate effect size (*d* = 0.50), $\alpha$ set at 0.05, and $\beta$ set at 0.80, to determine that at least 64 participants per group (control and intervention) were required to adequately power this investigation. Sample-size estimation was performed with the G*Power software, V 3.1.9.6 [59]. To account for attrition, screening failures, and possible re-injury, we will enroll a total of 180 runners to sufficiently power the protocol in order to adequately detect evident effects of the intervention.

This study’s goal is to measure the effectiveness of gait retraining in terms of longitudinal change in outcomes over 12 weeks. Descriptive statistics, including measures of central tendency and dispersion, will be calculated for demographic data. Frequency distributions will be estimated for categorical data. Separate 2-by-3 mixed-model analyses of variance (ANOVA) with group as the between-subjects factor (telehealth gait retraining plus standard physical therapy and standard physical therapy alone) and time as the repeated measure within-subjects factor (baseline, 8 weeks, and 12 weeks) will determine the effect of telehealth gait retraining on pain, self-reported function, and running biomechanics. Alpha will be set at 0.05 for all omnibus comparisons, which are the group*time interaction, the main effect for group (fixed factor), and the main effect for time (repeated measure). Planned pairwise comparisons will be performed to examine significant main effects for group using independent *t*-test and time using paired *t*-tests. Alpha for planned pairwise comparisons will be corrected using the Sidak’s correction to control for family-wise type I error. The Cohen *d* coefficient will be used to assess effect size between pairwise comparisons. Prior to performance of the ANOVAs, all outcome measures will be assessed for normality. The appropriate non-parametric statistical tests will be used for any non-normally distributed outcomes. All statistical analyses will be performed with the statistical package SPSS version 28 (IBM, Chicago, IL, USA).

Participants who are lost to follow-up due to failure to attend data collection sessions or non-adherence to the treatment protocol will be analyzed per the intention-to-treat concept [60]. Participants who only attend a baseline session but do not complete other data collection sessions will be considered to have not completed the allocated intervention and will be excluded. Additionally, participants who fail to submit a video for 2 consecutive weeks will be considered non-adherent to the treatment and will be excluded. Data for excluded participants will be replaced using multiple imputation [61].

The local study team will obtain the information necessary to complete the study case report forms (CRFs) from several sources including the participant’s medical record and clinical evaluations and directly from the participant. Investigators will collect study data directly from the study participant, from their attending provider, or, where applicable, from the participant’s medical record (i.e., relevant medical and treatment history and relevant clinical notes) and record on paper CRFs. Following each research visit, an investigator will review the paper CRFs for accuracy and completeness and then enter the collected non-personally identifiable data from the paper CRFs into REDCap. REDCap is an encrypted, access controlled, password-protected electronic data capture and management system housed on a Department of Defense (DoD) server and maintained by the Uniformed Services University Information Technology (USU IT) [62, 63]. No PII will be entered into REDCap.

With the exception of the Informed Consent Document, HIPAA Authorization, intake CRF, and electronic master list, all research data (both paper and electronic) will be identified using a unique study ID only, and not by the participant’s name, date of birth, DoD ID, or other protected identifier. Paper CRFs, consent forms, and HIPAA authorizations will be stored in a locked cabinet inside of a locked room and the coded electronic research data for this study will be stored in REDCap. All research data and forms (both paper and electronic) will only be accessible by authorized study staff, the local IRB, and applicable governmental agencies as part of their duties and in accordance with federal law.

To monitor regulatory compliance and safety, the local human protections administrator will audit the study once during active data collection. Study investigators will conduct bi-annual regulatory compliance and data quality checks. To ensure compliance, the regulatory binder will be checked for completeness and up to date documents. The binder will include the protocol and informed consent documents (all revisions), IRB approvals/correspondence, case report forms, and investigator documents. Study investigators will also conduct a data quality check of 10% of study records.

## Discussion

The purpose of this study is to determine the effectiveness of a telehealth gait retraining intervention on self-reported pain during and after running, self-reported function, and biomechanical risk factors for injury in military service members who present to a Military Health System physical therapy clinic with an overuse knee injury. While gait retraining to change foot strike pattern from a RFS to a NRFS has been shown to improve function, pain, and known biomechanical factors related to running knee injury [23, 25–27], most gait retraining interventions require multiple clinic visits [17] which limits feasibility in military physical therapy clinics. A telehealth gait retraining intervention might improve feasibility through decreased cost and time, but the effectiveness of a telehealth gait retraining intervention in patients with overuse knee injuries is not known. The results of this study may help determine the effectiveness and feasibility of a telehealth gait retraining intervention. Specifically, this study will determine if pain intensity differs when a standard physical therapy rehabilitation program is combined with either a return-to-run progression with telehealth gait retraining or a return-to-run progression without gait retraining after completion of the intervention and at 4 weeks post-intervention to assess retention of any potential benefits. Additionally, this study will determine if self-reported function and biomechanical risk factors for injury are different for the two groups after completion of the intervention and at 4 weeks post-intervention.

This study has several limitations and design constraints. The primary limitation is that the physical therapists and administering the gait retraining intervention and the participants will not be blinded to the treatment groups. However, the individuals assessing changes in foot strike pattern will be blinded to group allocation to minimize potential bias. Outside of the telehealth gait retraining intervention, physical therapists will prescribe individualized rehabilitation programs for each participant, creating potential inequities in the quantity and/or quality of interventions. This aspect of the trial was kept as pragmatic as possible to maximize generalizability. While the study was designed to be most generalizable to a military setting, study findings may also be transferable to patients with overuse knee pain in civilian populations.

### Supplementary Information

**Additional file 1.** Standard feedback cues.

**Additional file 2.** Lower leg and foot exercise program.

**Additional file 3.** SPIRIT 2013 Checklist: Recommended items to address in a clinical trial protocol and related documents*.

## Data Availability

The coded electronic research data for this study will be stored in Research Electronic Data Capture (REDCap), an encrypted, access-controlled, password-protected electronic data capture and management system housed on a DoD server and maintained by the Uniformed Services University of the Health Sciences Information Technology. Data from the study are available by email request to the lead author for the purpose of systematic review and meta-analysis.
