# Supplementary material for: The effectiveness of telehealth gait retraining in addition to standard physical therapy treatment for overuse knee injuries in soldiers: a protocol for a randomized clinical trial
Source: Trials. 2023 Oct 16;24:672. doi: 10.1186/s13063-023-07502-x (PMC10580615; doi:10.1186/s13063-023-07502-x)
Supplement: Supplementary file 2 — Additional file 2. Lower leg and foot exercise program. [file 13063_2023_7502_MOESM2_ESM.docx]

Appendix B - Lower Leg and Foot Exercise Program

***NOTE:*** *This lower leg and foot exercise program is designed to help improve the strength and flexibility of muscles surrounding the knee, ankle, and foot joints.*

1. **Calf Stretch**

Sets: 3 on each leg

Reps: 30 second hold


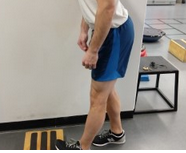


**DESCRIPTION:** Start with your toes up on a step and heel on the ground. Keep your other foot on the ground pointed towards the step. Hips should face forward. Make your shoulders big and slowly lean forward and move your butt backwards while keeping your stretch leg straightened out until a stretch is felt in the calf.

1. **Hamstring Stretch**

Sets: 3 on each leg

Reps: 30 second hold

Hamstring stretch


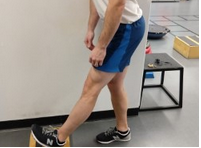


**DESCRIPTION:** Start with your heel on a step with your toes pointed up. Keep your other foot on the ground behind the step and pointed towards the step. Hips should face forward. Make your shoulders big and slowly lean forward and move your butt backwards while keeping your stretch leg straightened out until a stretch is felt in the hamstring.

1. **Eccentric Heel Lowering**

Sets: 3 on each leg

Reps: 12 to 15

Beginner Moderate Advanced


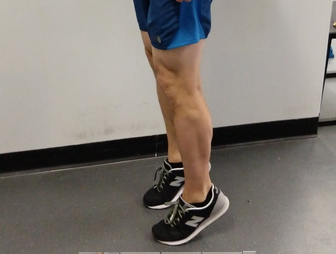

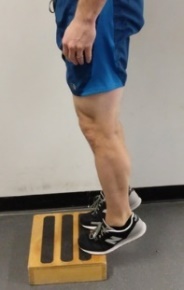

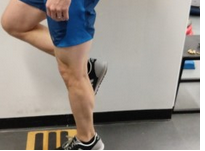


**DESCRIPTION:** Start on a level ground. Stand on the balls of your feet and raise both heels as high as you can. Slowly lower heels to ground with a 3 to 5 second count. To advance this exercise, perform as stated above but begin on a step and slowly lower your heels below the top of the step. An alternative for advanced heel lowers is to perform single leg on the ground and then advance to single leg on a step. Slow and controlled!

1. **Foot Doming**

Sets: 5 on each foot

Reps: 30 seconds

Relaxed foot Foot doming


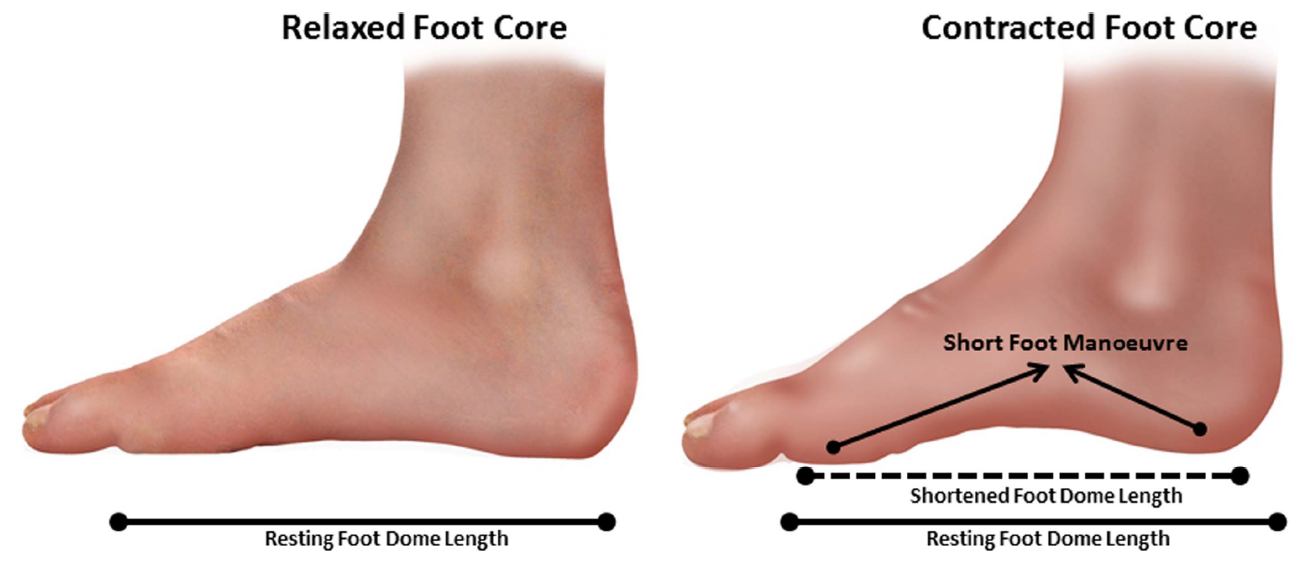
(Figure from McKeon, et al. 2014)

Example of foot doming


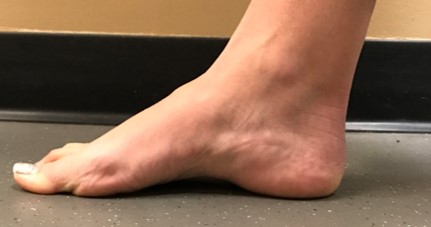


**DESCRIPTION:** Curl your toes into the ground. Raise the arch of your foot without lifting your heel off the ground. Hold for 30 seconds then repeat 5 times. Initially try this exercise seated. As you progress, complete this exercise while standing on both feet. After you have mastered this exercise in standing, progress to single leg standing while doming the foot. This exercise can be performed while sitting at your desk, watching TV, standing in line, etc…
